# Detection of zoonotic-borne parasites in *Rattus* spp. in Klang Valley, Malaysia

**DOI:** 10.14202/vetworld.2022.1006-1014

**Published:** 2022-04-22

**Authors:** Firdaus Mohd-Qawiem, Saulol Hamid Nur-Fazila, Raslan Ain-Fatin, Qian Hui Yong, Md Isa Nur-Mahiza, Abd Rahaman Yasmin

**Affiliations:** 1 Department of Veterinary Pathology and Microbiology, Faculty of Veterinary Medicine, Universiti Putra Malaysia, Serdang 43400, Selangor, Malaysia; 2 Department of Veterinary Laboratory Diagnostics, Faculty of Veterinary Medicine, Universiti Putra Malaysia, Serdang 43400, Selangor, Malaysia; 3 Laboratory of Vaccines and Immunotherapeutic, Institute of Bioscience, Universiti Putra Malaysia, Serdang 43400, Selangor, Malaysia

**Keywords:** ectoparasites, endoparasites, Klang Valley, rats, zoonosis

## Abstract

**Background and Aim:**

*Rattus* spp. are the most common animals capable of adapting to their environment. They can be reservoirs or vectors of diseases that facilitate the transmission of zoonotic-borne parasites to humans. Hence, a study on the detection of parasites in rat populations in urban areas is crucial to prepare for emerging zoonosis. Therefore, this study aims to identify blood parasites, ectoparasites, and helminths in *Rattus* spp. from wet markets located in Klang Valley, an urban area with a high-density human population.

**Materials and Methods:**

A total of 32 rats were trapped in several wet markets in Klang Valley, Malaysia. They were anesthetized for morphometric examination followed by exsanguination. Various parasitological techniques such as perianal tape test, simple flotation, direct examination of the intestine, and fecal smear were performed for intestinal parasite detection; hair plucking, skin scraping, and full body combing for ectoparasite identification; and blood smear, microhematocrit centrifugation, and buffy coat techniques for blood parasite detection.

**Results:**

The rats were identified as *Rattus rattus* (71.9%) and *Rattus norvegicus* (28.1%). The only blood protozoan found was *Trypanosoma lewisi*. The ectoparasites identified belonged to two broad groups, mites (*Laelaps* spp. and *Ornithonyssus* spp.) and fleas (*Xenopsylla cheopis*), known to be parasitic zoonotic disease vectors. The zoonotic intestinal parasites were cestodes (*Hymenolepis nana*), nematodes (*Nippostrongylus brasiliensis*, *Strongyloides* spp., *Trichuris* spp., *Capillaria* spp., and *Syphacia* spp.), and intestinal protozoa (coccidian oocysts and *Giardia* spp.). Microscopic images showing *Giardia* spp. are the first report of this organism in rats in Malaysia.

**Conclusion:**

Rats caught in this urban area of the Klang Valley harbor parasites can pose a potential zoonotic threat to humans, raising public health concerns because of their proximity to densely populated urban areas.

## Introduction

Rats, members of the rodent family Muridae, are extremely successful and dominant species worldwide because of their ability to adapt to various environments of urbanization, cultivated land, forest, and others. A study conducted by Chakma *et al*. [1] mentioned that rats are the most destructive agricultural pests and vectors of zoonotic diseases worldwide, such as plague, leptospirosis, leishmaniasis, and Hantavirus. Because of the physiological similarity between rats and humans, their huge diversity, and the fact that some species of rats have adapted to living in close contact with humans, rats play a crucial role as reservoirs and vectors for zoonotic diseases [2].

Rats are a reservoir host for many zoonotic pathogens, including parasitic diseases such as hymenolepiasis, trichinellosis, echinococcosis, capillariasis, and toxoplasmosis [3]. Rodents transmit more than 60 infectious diseases to humans, directly and indirectly, or, in some cases, serve as a crucial intermediate host in maintaining the life cycle of the parasite [2]. The transmission of parasites to the host can occur in several parasitic life cycles, such as spores, eggs, cysts, and juveniles [4]. They pose serious public health problems by transmitting zoonotic diseases to humans.

Rats live in high density, and their proximity to humans has allowed the transmission of infection to the population more rapidly in major cities worldwide [5]. Therefore, the rapidly changing urban environment implicates the need to overcome emerging zoonosis [6].

Rats commonly seen in Malaysia belong to two species; the common house rat (*Rattus rattus diardii*) and the Norway rat (*Rattus norvegicus*) [7]. A wide variety of parasites has been found in rats in various areas in Malaysia [8,9]. Although studies on parasites have been conducted in wild and urban rats in Malaysia, no recent study has been found on the detection of parasites in rat populations residing in densely populated regions of Malaysia, especially in wet market areas that serve as a hub of goods distributors for retailers and consumers.

This study aims to identify parasites in rats in wet markets around the Klang Valley, in which the wholesale markets in nearby residential areas become favorable food sources for rats. This study allows one to gather useful information to assess the potential transmission of zoonotic parasites to humans.

## Materials and Methods

### Ethical approval

This study was approved by Universiti Putra Malaysia (UPM) Institutional Animal Care and Usage Committee (IACUC) with an approval code of UPM/IACUC/AUP-U017/2019.

### Study period and location

The study was conducted from July to September 2019. The locations for the rat trapping and collection were Pasar Borong Selangor, Pasar Seri Kembangan Selangor, and Pasar Borong Kuala Lumpur, Klang Valley, Malaysia. The samples were processed at Veterinary Parasitology Laboratory, Faculty of Veterinary Medicine, UPM.

### Animal sampling

A total of 32 rats were collected through cage traps placed at several locations of wet markets around Klang Valley, Malaysia. They were anesthetized with diethyl ether at 100 mg/mL [10]. Under general anesthesia, 3-5 mL of blood was withdrawn through intracardiac puncture followed by terminal exsanguination for euthanasia. They were brought to the Post-Mortem Laboratory, Faculty of Veterinary Medicine, UPM, for further examinations. Protective clothing such as laboratory coats, double-layer gloves, and disposable N95 face masks was worn when handling the animals to avoid the transfer of diseases. The habit of handwashing after contact with animals was inculcated. The postmortem room was kept clean according to the standard cleaning method. Equipment and dissecting kits were disinfected after use. The carcasses were disposed of as bioclinical waste using a yellow biohazard bag.

### Identification of rats

**Figure-1:**
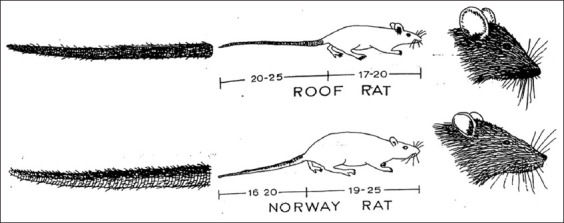
Comparison of *Rattus rattus* (roof rat) and *Rattus norvegicus* (Norway rat) (Adapted from Manual of MSPTM Rodent Workshop Program, 2003).

All captured rats were subjected to morphometric examinations that included the length, color of the fur, and morphological features such as the snout, ears, eyes, body size, and tail for species identification. The sex and age of the rats were also recorded. Figure-1 illustrates the comparison of *R. rattus* and *R. norvegicus* species.

### Examination of blood parasites

The blood samples were subjected to thin and thick blood smears and microhematocrit centrifugation. Thin and thick blood smears were stained with Giemsa stain to detect blood protozoans [6], whereas the microhematocrit centrifugation was performed to identify the presence of trypanosomes [11].

### Examination of ectoparasites

**Figure-2:**
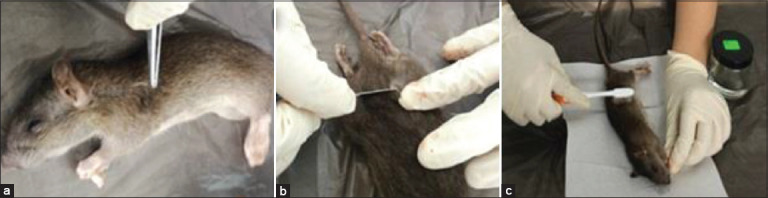
Demonstration of ectoparasites examination by (a) fur pluck, (b) skin scraping, and (c) fur combing techniques.

Ectoparasites were detected by fur plucking, skin scraping, and fur combing techniques [4,12,13], as shown in Figure-2. If present, fur plucking and skin scraping tests were performed in the scapular, axillary, inguinal, dorsal rump, cervical, and lesions regions. The collected ectoparasites were placed onto glass slides before the examination, and the fur of each rat was also combed with a fine toothbrush. The ectoparasites were picked and placed in the sample bottles containing 75% alcohol. Then, they were mounted with Hoyer’s medium for further observation under a light microscope at 80×, 200×, and 400×.

### Examination of intestinal parasites

Intestinal helminths and protozoa were identified using a perianal tape test, fecal smear, simple flotation, and direct examination of intestinal tract techniques [13]. A perianal tape test was used to examine the helminth eggs, and a fecal smear test was performed to detect intestinal protozoa. The fecal flotation method was performed to identify the coccidian oocysts, helminth ova, and their larval stages. All collected helminths were preserved in 75% alcohol. The nematodes were fixed and cleared with lactophenol as temporary mounts. Cestodes were stained with carmine acetoalum and mounted in dibutylphthalate polystyrene xylene for microscopic examination under a light microscope (Zeiss PrimoStar, Germany) according to morphological characteristics at 40× and 400×.

## Results

In this study, two different species of rats were identified as *R. rattus* (23; 71.9%) and *Rattus norvegicus* (9; 28.1%) based on length, fur color, and features as previously reported [14]. *R. rattus* has a pointed snout, big eyes, big ears, a slender body, and a unicolor tail longer than the body. *R. norvegicus* has a slanted snout, small eyes, small ears, a sturdy body, and a bicolor tail shorter than the body. Male hosts (18; 56.3%) were more commonly caught than female hosts (14; 43.8%) for this study. As per the general age group, juveniles were more frequent at 56.3% compared with adults (43.8%). All captured rats were positive for parasites with at least one parasite or up to seven parasites in a single host. Table-1 summarizes the host captured relative to species, sex, age, and the number of hosts infected. The distribution of the parasites found in the rat population is shown in Table-2.

**Table 1:** Species of rats infected with different types of parasites according to sex and age.

Rat species	Host sex (%)		Host age (%)		Number of hosts infected (percentage %)			Total number of captured rats (percentage %)
		
	M	F	A	J	Blood parasites	Ectoparasites	Intestinal parasites	
*Rattus rattus*	12 (52.2)	11 (47.8)	12 (52.2)	11 (47.8)	2 (8.7)	17 (73.9)	23 (100)	23 (71.9)
*Rattus norvegicus*	6 (66.7)	3 (33.3)	2 (22.2)	7 (77.8)	0	8 (88.9)	9 (100)	9 (28.1)
Total	18 (56.3)	14 (43.8)	14 (43.8)	18 (56.3)	2 (6.3)	25 (78.1)	32 (100)	32

M=Male, F=Female, A=Adult, J=Juvenile

**Table 2:** Distribution of the parasites found in the rat population.

Parasites	No. of rats infected rats (percentage %) (N=32) at 95%CI	

	Zoonotic	Non-zoonotic
Blood parasites	*Trypanosoma lewisi* 2 (6.3%)	
Ectoparasites		
Mites	*Ornithonyssus* spp. 17 (53.1%)	*Laelaps* spp. 24 (75%)
Fleas	*Xenopsylla cheopis* 14 (43.8%)	
Intestinal parasites		
Nematodes	*Strongyloides* spp. 26 (81.3%)	
	*Nippostrongylus brasiliensis* 17 (53.1%)	
	*Capillaria* spp. 2 (6.3%)	
	*Trichuris* spp. 1 (3.1%)	
	*Syphacia* spp. 1 (3.1%)	
Cestodes	*Hymenolepis nana* 15 (46.9%)	
Protozoa	*Eimeria* spp. 9 (28.1%)	
	*Giardia* spp. 1 (3.1%)	

N=Total number of captured rats, CI=Confidence interval

### Identification of blood parasites

**Figure-3:**
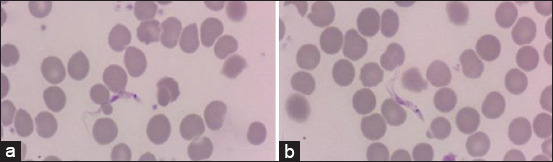
(a and b) Presence of *Trypanosoma lewisi* in *Rattus rattus* at 2000×.

*Trypanosoma lewisi* was observed in two rats of *R. rattus*, but none was observed in *R. norvegicus*. As previously described by Kamaruzaman *et al*. [15], this parasite has a pointed posterior end with a medium-sized kinetoplast, a well-undulated membrane, and a free anterior flagellum, as illustrated in Figure-3.

### Ectoparasites examination

**Figure-4:**
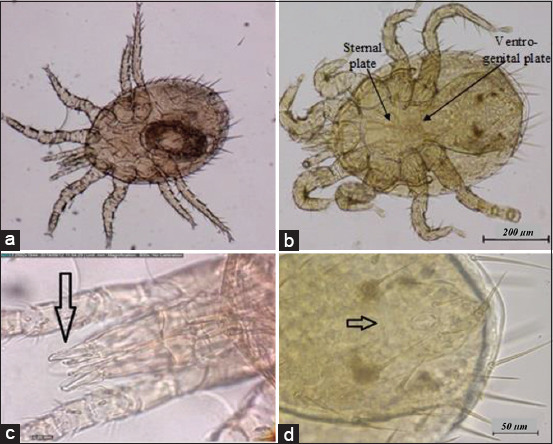
Presence of *Laelaps* spp. (a) *Laelaps* spp. at 80×, (b) *Laelaps* spp. 40×. *Laelaps* spp. has a semi-rectangular sternal plate, slightly longer rather than wider, elongated, and flask-shaped of the ventrogenital plate, (c) chelicera of *Laelaps* spp. (arrow) at 800×, and (d) anal plate of *Laelaps* spp. (arrow) at 400×.

**Figure-5:**
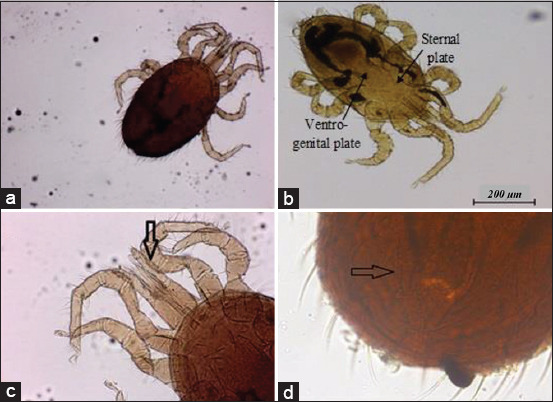
Presence of *Ornithonyssus* spp. (a) *Ornithonyssus* spp. at 80×, (b) *Ornithonyssus* spp. at 40×. It has a rectangular sternal plate, an elongated, and a finger-like ventrogenital plate, (c) palp of *Ornithonyssus* spp. (arrow) at 400×, and (d) anal plate of *Ornithonyssus* spp. (arrow) at 800×.

**Figure-6:**
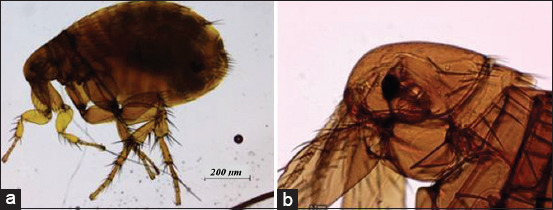
Presence of *Xenopsylla cheopi*s. (a) *Xenopsylla cheopis* at 40×and (b) head of *Xenopsylla cheopi*s with light amber in color and lack of both genal and pronotal combs at 200×.

Twenty-five out of 32 rats (78.1%) were infected with ectoparasites. Each infected rat harbored at least one to three different ectoparasites (34.4%). *R. norvegicus* and *R. rattus* were infected with ectoparasites at 88.9% and 73.9%, respectively. Two mite species, *Laelaps* spp. and *Ornithonyssus* spp., and a single species of flea, *Xenopsylla cheopis* (Figures-4-6), accounted for 75.0%, 53.1%, and 43.8%, respectively.

### Intestinal parasite identification

**Figure-7:**
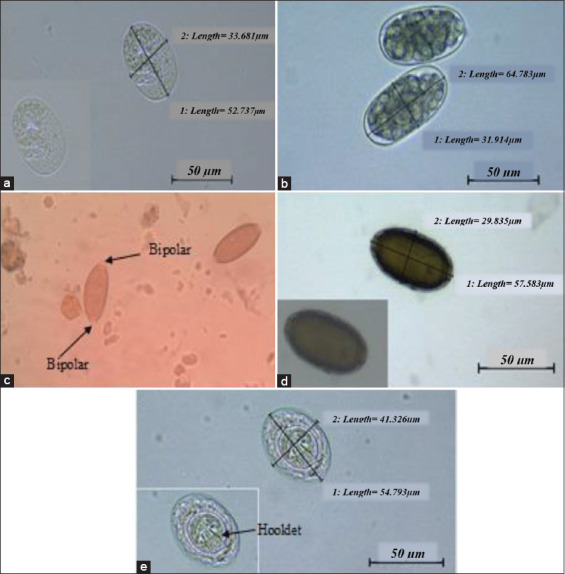
Helminths ova at 400×. (a) *Strongyloides* spp. ova are an embryonated oval shape with a thin and transparent shell, (b) *Nippostrongylus brasiliensis* ova are ellipsoidal and thin-shelled, (c) *Trichuris* spp. ova are barrel-shaped with three layers and two protruding bipolar plugs, (d) *Capillaria* spp. ova are similar to *Trichuris* spp. ova, but the shells appeared to be more striated, and (e) *Hymenolepis nana* ova shapes were oval, with three pairs of hooklets and prominent polar thickenings with polar filaments found between the developing embryo and the eggshell.

All rats were infected with at least one or up to a maximum of four genera of intestinal parasites. In this study, eight genera of parasites were found; five genera of nematodes of *Strongyloides* spp. (81.3%), *Nippostrongylus brasiliensis* (53.1%), *Capillaria* spp. (6.3%), *Trichuris* spp. (3.1%), and *Syphacia* spp. (3.1%) and one cestode species of *Hymenolepis nana* (46.9%). Intestinal protozoans of *Giardia* spp. (3.1%) and coccidian oocyst (28.1%) were also found. The nematode and cestode eggs were identified based on the morphology of the eggs obtained from a simple flotation test, as shown in Figure-7. However, the egg of *Syphacia* spp. was not seen in this study.

**Figure-8:**
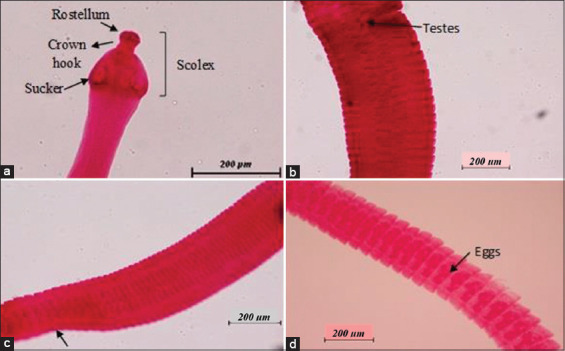
*Hymenolepis nana* at 400×. (a) The scolex of *H. nana* has small, globular, and cup-like scolex at the anterior end, with four suckers, an armed retractile rostellum, and crown hooks, (b) mature proglottid has visible testes, (c) immature proglottid is slightly narrow (arrow), and (d) gravid proglottid is rounded and filled with eggs.

**Figure-9:**
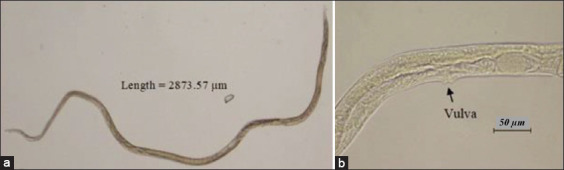
*Strongyloides* spp. helminth. (a) *Strongyloides* spp. is filiform in shape, with a narrow cylindrical esophagus that extends a quarter length of the nematodes at 40× and (b) vulva of the female *Strongyloides* spp. at the posterior third of the body at 400×.

**Figure-10:**
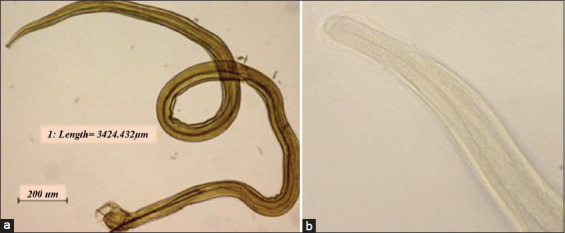
*Nippostrongylus brasiliensis* helminth. (a) *N. brasiliensis* and bursa are filiform and reddish in color at 40× and (b) mouthpart of *N. brasiliensis* at 400×.

**Figure-11:**
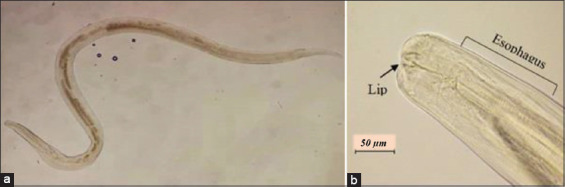
Presence of *Syphacia* spp. helminth. The male’s bursa has asymmetry lateral lobes and small dorsal lobes with the presence of spicules and gubernaculum. (a) *Syphacia* spp. at 40× and (b) mouth of *Syphacia* spp. has three distinct lips without a buccal capsule, and its esophagus has pre-bulbar swelling and a posterior globular bulb at 400×.

The adult cestode of *H. nana* was obtained in this study. Each proglottid has both male and female reproductive organs, making *H. nana* hermaphroditic. The mature proglottids are wider than the immature proglottids with the testes visible, and the gravid proglottids are filled with eggs, as shown in Figure-8. Other than that, the adult nematodes identified were *Strongyloides* spp., *N. brasiliensis*, and *Syphacia* spp., as shown in Figures-9-11, respectively.

**Figure-12:**
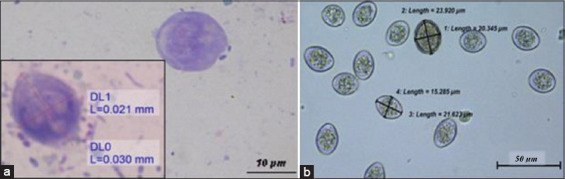
*Giardia* spp. protozoa and coccidian oocysts. (a) *Giardia* spp. at 2000× and (b) unsporulated coccidian oocysts appear rounded to oval shape with a thin wall at 400×.

The intestinal protozoans found in the feces of rats are *Giardia* spp. and *Eimeria* spp. oocysts, as shown in Figure-12. *Giardia* spp. was seen in the fecal smear stained with Giemsa stain. The trophozoites are bilaterally symmetric and have a pyriform shape with two anterior nuclei and two slender axostyles, but the flagellates were not seen (Figure-12a).

## Discussion

This study aims to identify parasites in *Rattus* spp. in wet markets around the Klang Valley. Rats harbor different types of parasites, some of which are zoonotic and can cause a serious impact on both animals and humans. Since rats live near humans, it is crucial to understand the possible implications for humans and the environment imposed by the parasites harbored in these rats. The rat population in the urban area was maintained by the high density of residential areas around the market, continuous human activities, ample food availability, and inappropriate waste management in the market. These factors contribute to the exposure of rodents to the transmission of disease to humans.

In the study, two species of rats, *R. rattus* and *R. norvegicus*, were identified, in agreement with a previous study conducted by Zain *et al*. [14]. However, the other study recorded up to five species of rats captured in Kuala Lumpur, namely, *R. rattus diardii* (Malayan black rat), *R. norvegicus* (Norway rat), *Rattus argentiventer* (ricefield rat), *Rattus tiomanicus* (Malaysian field rat), and *Rattus exulans* (Polynesian rat) [8], most likely because of their significant presence in the various selected study areas.

*T. lewisi* is the only blood parasite detected in the present study. Similarly, the previous studies of Shafiyyah *et al*. [8] and Premaalatha *et al*.[9] also detected *T. lewisi* infection among pest rats around Kuala Lumpur. Although it is specific to a single vertebrate host of the *Rattus* genus, it could be transmitted by a wide variety of flea vectors such as *X. cheopis*, *Nosopsyllus fasciatus*, *Ctenocephalides canis*, and *Ctenocephalides felis* [6]. Rats are infected with *T. lewisi* primarily through oral route transmission, most commonly by flea ingestion during fur grooming [16]. Although *T. lewisi* is considered non-pathogenic in rodents, it has been documented to cause disease in humans on rare occasions in a suitable environment, host, and organism [8]. Therefore, it is necessary to prepare for emerging zoonosis.

Ectoparasites such as *Laelaps* spp., *Ornithonyssus* spp., and *X. cheopis* reported in this study are also supported by a recent finding of Tijjani *et al*. [17] in student hostels at UPM. In a separate study by Priscilla *et al*. [18], *Laelaps* spp. was also detected in Recycle Energy Sdn Bhd Semenyih, Ladang Pertanian Bersepadu, UPM, and the Sri Serdang housing area, Selangor. These buildings are only 10-30 km from the study areas. *R. norvegicus* had a higher percentage of ectoparasite infestation, probably because they live in a larger group that has a greater chance of their transmission than *R. rattus*. The sex-related difference between rats observed in the present study is possibly by chance. It could be attributed to the males who have a wider home range and are more susceptible to ectoparasite infection. Rats are actively foraging and can contribute to the increased number of ectoparasite infestations [19].

Fleas found in the study are known to transmit bubonic plague from rodents to humans that are endemic in several regions in Southeast Asia, with regular outbreaks among humans [17]. In the present study, 43.75% of the captured rats were recorded among wild rats in the presence of *X. cheopis*. According to a previous finding by Dennis *et al*. [20], when the number of *X. cheopis* in rats increases above a certain level, it represents a potentially dangerous situation. Thus, preventive measures should be taken to decrease the risk of human cases and plague epizootics.

The mite species of *Laelaps nuttali*, *Laelaps echidinus* (spiny rat mites), and *Ornithonyssus bacoti* (tropical rat mites) are common ectoparasites found in rats and possess medical importance because of the transmission of diseases to humans. Although *Laelaps* spp. and *Ornithonyssus* spp. were detected in the present study, identification of *Laelaps* spp. was not made until the species level. *O. bacoti* bites humans as an accidental host, causing mite dermatitis and possibly transmitting filariasis to humans [17]. In 1974, the first confirmed case of *O. bacoti* caused dermatitis was reported in Malaysia [21]. *Laelaps echidninus* found in *Rattus* spp. sometimes causes skin irritation in humans [22].

In this present study, all rats were infected with intestinal parasites regardless of species, sex, and age of the hosts. Therefore, they are equally contaminated with helminth ova and protozoa oocysts, probably because of the unhygienic environment on the premises. *H. nana* (dwarf tapeworm) was the only cestode detected in this study. Other researchers reported the presence of *H. nana* in their studies of wild rats in Kuala Lumpur [3,9] and Ulu Gombak [23]. The smallest tapeworm could also infect humans, causing hymenolepiasis [14] with clinical signs of restlessness, irritability, diarrhea, and abdominal pain in humans [9].

In the present study, five species of nematodes were identified up to the genus level, namely, *Strongyloides* spp., *Nippostrongylus* spp., *Capillaria* spp., *Trichuris* spp., and *Syphacia* spp. Conversely, a study by Roberts and Janovy [3] recorded additional nematodes of *Heterakis* spp., *Physaloptera* spp., *Pterogodermati*s spp., *Gongylonema neoplasticum*, and *Rictularia tani*, possibly because of the larger sample size and broad selection areas. *Strongyloides* spp. is the most common intestinal nematodes detected in this study, previously characterized as *S. ratti* and *S. venezuelensis* [24]. *Nippostrongylus* spp. is the second common nematode detected in this study, as also reported among the rat population in Kuala Lumpur [8,9], and Carey Island [25]. Other nematodes such as *Capillaria* spp., *Trichuris* spp., and *Syphacia* spp. were less likely to infect rats. As a zoonotic potential, *Capillaria* spp. causes hepatic capillariasis and can affect rats but is rarely seen in humans. The clinical features of liver capillariasis range from acute to subacute hepatitis with eosinophilia. However, it may cause death from severe liver damage with secondary bacterial infection [8].

Intestinal protozoa such as *Eimeria* spp. and *Giardia* spp. were also discovered in this study. *Eimeria* species were not identified, as oocyst sporulation was not done because of the limited amount of rat urine collected. Since there is currently no record of *Giardia* spp. from microscopic examination, this current finding is the first report of microscopic images of *Giardia* spp. found in rats in Malaysia. Meanwhile, Tan *et al*. [26] has reported the zoonotic protozoa of genotype B of *Giardia duodenalis* in urban rodents in Malaysia that were detected molecularly through a polymerase chain reaction.

Rats transmitted zoonotic parasitic diseases present in the study area, most likely due to the proximity of these rats in an urban area with a high density of human population, as previously reported by Galán-Puchades *et al*. [27]. Coupled with the desired environment in wet markets as well as poor sanitation and hygiene of personnel, it could be a disastrous situation if an outbreak of human disease was to occur.

## Conclusion

All rats captured in Klang Valley were infected with zoonotic parasites, including cestode (*H. nana*), nematodes (*N. brasiliensis*, *Strongyloides* spp., *Trichuris* spp., *Capillaria* spp., and *Syphacia* spp.), ectoparasites (*Ornithonyssus* spp. and *X. cheopis*), and blood parasites (*T. lewisi*) that potentially cause illnesses to humans. However, *Laelaps* spp. is the only parasite found not to be zoonotic in this study. Therefore, precautionary steps need to be taken to prevent the spread of diseases from rat populations to humans in urban areas of Klang Valley. Limitations of the study are the small sample size and limited diagnostic techniques used in diagnosing parasites. It is recommended to perform other techniques such as formal-ether sedimentation technique to identify helminths ova and coccidia oocysts, and molecular method for the identification of the parasite up to species level since certain species of parasites characterization by morphological features was not possible. Moreover, the sample size should be increased, and the association of sex and age of the rats and the infection of the parasite could be conducted in future studies.

## Authors’ Contributions

SHN: Designed the study. FM: Drafted the manuscript. QHY: Performed the experiment, sampling, and laboratory works. RA: Contributed to the sampling and laboratory works. SHN, ARY, and MIN: Critically revised the manuscript. All authors read and approved the final manuscript.

## References

[1] Chakma N, Sarker N, Belmain S, Sarker S, Aplin K, Sarker S (2018). New records of rodent species in Bangladesh:Taxonomic studies from rodent outbreak areas in the Chittagong hill tracts. Bangladesh J. Zool.

[2] Belmain S.R (2006). Rats and Human Health in Africa.

[3] Paramasvaran S, Sani R.A, Hassan L, Hanjeet K, Krishnasamy M, John J, Santhana R, Sumarni M.G, Lim K.H (2009). Endo-parasite fauna of rodents caught in five wet markets in Kuala Lumpur and its potential zoonotic implications. Trop. Biomed.

[4] Roberts L.S, Janovy J (2006). Foundations of Parasitology, in Parasitic Insect:Mallophaga and Anoplura Lice.

[5] Himsworth C.G, Parsons K.L, Jardine C, Patrick D.M (2013). Rats, cities, people, and pathogens:A systematic review and narrative synthesis of literature regarding the epidemiology of rat-associated zoonoses in urban centers. Vector Borne Zoonotic Dis.

[6] Alias S.N, Sahimin N, Edah M.A, Mohd-Zain S.N (2014). Epidemiology of blood parasitic infections in the urban rat population in peninsular Malaysia. Trop. Biomed.

[7] Liat L.B (2015). The field rats and field mouse in Malaysia and Southeast Asia. UTAR Agri. Sci. J.

[8] Shafiyyah C.O.S, Jamaiah I, Rohela M, Lau Y.L, Aminah F.S (2012). Prevalence of intestinal and blood parasites among wild rats in Kuala Lumpur, Malaysia. Trop. Biomed.

[9] Premaalatha B, Chandrawathani P, Jamnah O, Farah Haziqah M.T, Tan P.S, Tharshini J, Ikhmal S.N, Ramlan M, Norhafiza H, Khadijah S, Mariappan C (2018). A survey of parasitic infections in wild rats from urban areas in Kuala Lumpur, Malaysia. Malays. J. Vet. Res.

[10] Bossi D.E, Linhares A.X, Bergallo H.G (2002). Parasitic arthropods of some wild rodents from Jureia-ltatins Ecological Station, State of Sao Paulo, Brazil. Mem. Inst. Oswaldo. Cruz.

[11] Chagas C.R.F, Binkiene R, Ilgunas M, Lezhova T, Valkiunas G (2020). The buffy coat method:A tool for detection of blood parasites without staining procedures. Parasit. Vectors.

[12] Madinah A, Fatimah A, Mariana A, Abdullah M.T (2011). Ectoparasites of small mammals in four localities of wildlife reserves in peninsular Malaysia. Southeast Asian J. Trop. Med. Public Health.

[13] Parkinson C.M, O'Brien A, Albers T.M, Simon M.A, Clifford C.B, Pritchett-Coming K.R (2011). Diagnosis of ecto-and endoparasites in laboratory rats and mice. J. Vis. Exp.

[14] Zain S.N.M, Behnke J.M, Lewis J.W (2012). Helminth communities from two urban rat populations in Kuala Lumpur, Malaysia. Parasit. Vectors.

[15] Kamaruzaman I, Ting H.W, Mokhtar M, Yuan Y.K, Shah A, Hamid F, Zalati C, Shaharulnizim N, Reduan M, Abu-Bakar L (2021). First case report on molecular detection of *Trypanosoma lewisi* in an urban rat in Kelantan, Malaysia:An accidental finding. J. Adv. Vet..

[16] Desquesnes M, Yangtara S, Kunphukhieo P, Jittapalapong S, Herder S (2016). Zoonotic trypanosomes in South East Asia:Attempts to control *Trypanosoma lewisi* using human and animal trypanocidal drugs. Exp. Parasitol.

[17] Tijjani M, Majid R.A, Abdullahi S.A, Unyah N.Z (2020). Detection of rodent-borne parasitic pathogens of wild rats in Serdang, Selangor, Malaysia:A potential threat to human health. Int. J. Parasitol. Parasites Wildl.

[18] Priscilla D, Jambari H.A, Meenakshii N (2015). Prevalence of mouse and rat parasites in resource recovery plants, farms and housing areas of Southern Selangor:Implication for public health. Pertanika J. Trop. Agric. Sci.

[19] Soliman S, Marzouk A.S, Main A.J, Montasser A.A (2001). Effect of sex, size, and age of commensal rat hosts on the infestation parameters of their ectoparasites in a rural area of *Egypt. J. Parasitol*.

[20] Dennis D.T, Gage K.L, Gratz N, Poland J.D, Tikhomirov E (1999). Plague Manual:Epidemiology, Distribution, Surveillance and Control.

[21] Nadchatram M, Ramalingham S (1974). Dermatitis caused by *Ornithonyssus bacoti* (Hirst 1913). Southeast Asian J. Trop. Med. Public Health.

[22] Ng Y.L, Hamdan N.E.S, Tuen A.A, Mohd-Azlan J, Chong Y.L (2017). Co-infections of ectoparasite species in synanthropic rodents of western Sarawak, Malaysian Borneo. Trop. Biomed.

[23] Paramasvaran S, Krishnasamy M, Lee H.L, John J, Lokman H, Naseem B.M, Rehana A.S, Santhana R.J (2006). Helminth infections in small mammals from Ulu Gombak Forest Reserve and the risk to human health. Trop. Biomed.

[24] Viney M, Kikuchi T (2017). *Strongyloides ratti* and *S. venezuelensis* rodent models of *Strongyloides* infection. Parasitology.

[25] Nursyazana M.T, Mohd-Zain S.N, Jeffery J.M (2013). Biodiversity and macroparasitic distribution of the wild rat population of Carey Island, Klang. Trop. Biomed.

[26] Tan T.K, Low V.L, Ng W.H, Ibrahim J, Wang D, Tan C.H, Chellappan S, Lim Y.A.L (2019). Occurrence of zoonotic *Cryptosporidium* and *Giardia duodenalis* species/genotypes in urban rodents. Parasitol. Int.

[27] Galán-Puchades M.T, Trelis M, Sáez-Durán S, Cifre S, Gosálvez C, Sanxis-Furió J, Pascual J, Bueno-Marí R, Franco S, Peracho V, Montalvo T, Fuentes M.V (2021). One health approach to zoonotic parasites:Molecular detection of intestinal protozoans in an Urban Population of Norway Rats, *Rattus norvegicus*, in Barcelona, Spain. Pathogens.

